# Electroacupuncture Ameliorates Cyclophosphamide‐Induced Ovarian Impairment in Rats With Diminished Ovarian Reserve and is Associated With Th17/Treg‐Related Immune Modulation

**DOI:** 10.1155/mi/1067286

**Published:** 2026-03-28

**Authors:** Xiaoyu Zhang, Zhanyu Lin, Ruixin Liu, Zhengqi Guo, Kexiang Wang, Yuxia Ma

**Affiliations:** ^1^ Department of Acupuncture and Massage College, Shandong University of Traditional Chinese Medicine, Jinan, 250355, Shandong, China, sdutcm.edu.cn; ^2^ College of Traditional Chinese Medicine, Shandong University of Traditional Chinese Medicine, 4655 University Road Changqing District, Jinan, 250355, Shandong, China, sdutcm.edu.cn; ^3^ College of Health, Shandong University of Traditional Chinese Medicine, 4655 University Road Changqing District, Jinan, 250355, Shandong, China, sdutcm.edu.cn

**Keywords:** diminished ovarian reserve, electroacupuncture, granulosa cells, immune modulation, Th17/Treg

## Abstract

**Background:**

Diminished ovarian reserve (DOR) is a major challenge in reproductive medicine, especially with delayed childbearing. Current treatments show limited efficacy and side effects. Electroacupuncture (EA), a multitarget nonpharmacological therapy, may protect ovarian function and regulate immune balance.

**Objectives:**

This study investigated the protective effects of EA and its potential mechanisms in cyclophosphamide (CTX)‐induced DOR in rats, with a focus on apoptosis‐related changes in granulosa cells and T helper 17 (Th17)/regulatory T (Treg)‐related immune modulation.

**Methods:**

A CTX‐induced DOR rat model was treated with EA at CV4 (Guanyuan) and CV6 (Qihai), with sham EA and normal groups as controls. Estrous cycle, ovarian indices, serum hormones, and cytokines were assessed. Ovarian morphology, follicle counts, apoptosis, and protein expression were evaluated by histology, TUNEL, western blotting (WB), immunohistochemistry (IHC), and immunofluorescence (IF). Splenic Treg/Th17 cells were analyzed by flow cytometry, and RNA sequencing identified EA‐regulated pathways.

**Results:**

EA improved estrous cyclicity, ovarian morphology, and follicular development; improved follicle‐associated marker expression; and reduced elevated FSH and LH levels in CTX‐induced DOR rats. AMH and E2 showed upward trends after EA treatment, but these changes did not reach statistical significance. EA reduced TUNEL positivity, restored Ki67 expression, and favorably regulated the Bcl‐2/Bax axis, while cleaved caspase‐3 remained elevated and was not significantly altered. EA was also associated with normalization of Th17/Treg‐related immune indices in the spleen, serum, and ovary, including reduced IL‐6, IL‐17A, IL‐1β, TNF‐α, and RORγt, and increased FOXP3, IL‐10, and TGF‐β1. Transcriptomic analysis revealed enrichment of immune‐related pathways, consistent with the functional findings.

**Conclusion:**

EA ameliorated CTX‐induced ovarian injury and was associated with improved follicular development, attenuation of apoptosis‐related changes, and Th17/Treg‐related immune modulation. These findings support a potential ovarian‐protective and immunoregulatory role of EA in DOR, although further studies are required to verify endocrine efficacy and mechanistic causality.

## 1. Introduction

Diminished ovarian reserve (DOR) is a growing challenge in reproductive medicine, particularly among older women [1]. Its prevalence is rising, with 31.6% of infertile patients undergoing assisted reproductive technology (ART) diagnosed with DOR, and about 25% of cases associated with chemotherapy or radiotherapy [2–4]. Without timely intervention, DOR often progresses to premature ovarian insufficiency [5]. Current treatments, including ART, coenzyme Q10, and hormone therapy, are limited by side effects and unsatisfactory long‐term efficacy, underscoring the need for safer and more effective strategies [6, 7].

At the mechanistic level, accumulating evidence indicates that granulosa cell dysfunction plays a central role in DOR progression. As the primary somatic component of ovarian follicles, granulosa cells regulate steroidogenesis, cytokine secretion, and oocyte maturation; their apoptosis leads to impaired folliculogenesis and accelerated follicular atresia [8, 9]. Immune dysregulation, particularly an imbalance between T helper 17 (Th17) cells and regulatory T (Treg) cells, has also emerged as a key pathological driver [10, 11]. Excessive Th17‐mediated inflammation and reduced Treg‐mediated immune tolerance disrupt ovarian homeostasis, contributing to granulosa cell injury and diminished follicular development [12–14].

Electroacupuncture (EA), a modern adaptation of traditional acupuncture with controllable stimulation parameters, has been increasingly studied for its regulatory effects on endocrine and immune systems [15, 16]. EA has shown potential in delaying ovarian aging, promoting natural conception, and improving ART outcomes [17, 18]. Acupoint selection analyses revealed the Ren meridian as the most frequently targeted in DOR treatment [15]; our real‐world data further supported its association with uterine–ovarian disorders and led to an optimized EA protocol [19]. Classical literature also highlights CV4 (Guanyuan) and CV6 (Qihai) as the most commonly used Ren meridian acupoints, which were selected in this study [20].

Despite growing evidence, it remains unclear whether EA improves DOR through coordinated modulation of immune homeostasis and attenuation of granulosa‐cell injury. To address this gap, the present study investigated the therapeutic effects of EA and its potential mechanisms in a cyclophosphamide (CTX)‐induced rat model of DOR, focusing on apoptosis‐related changes in granulosa cells and Th17/Treg‐related immune regulation.

## 2. Materials and Methods

### 2.1. Animal Experiments

Six‐week‐old female Sprague–Dawley (SD) rats (160–180 g) were purchased from Beijing Vital River Laboratory Animal Technology Co., Ltd. All experimental procedures were performed in accordance with the Guide for the Care and Use of Laboratory Animals (NIH, Bethesda, MD, USA) and were approved by the Institutional Animal Care and Use Committee of Shandong University of Traditional Chinese Medicine (Approval Number SDUTCM20250325001). Animals were housed in a specific pathogen‐free (SPF) facility under controlled environmental conditions (22 ± 2°C, 50% ± 5% humidity, 12 h light/dark cycle) with free access to standard chow and water. After 7 days of acclimation, sexual maturity was confirmed by checking for vaginal opening and assessing estrous cyclicity via vaginal smear cytology. Only rats that had entered sexual maturity and exhibited regular estrous cycles were then randomized into groups:

NOR normal ovarian reserve group): intraperitoneal injection of normal saline.
DOR: intraperitoneal CTX (Baxter Oncology GmbH, Germany) to induce DOR.
EA: CTX administration followed by EA treatment.
SA (Sham EA): CTX administration followed by sham EA.

**Figure 1:**
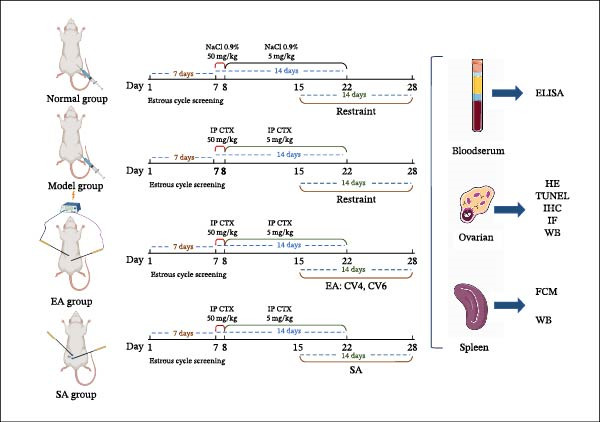
Experimental workflow. Schematic overview of the study design, including the establishment of the cyclophosphamide‐induced DOR rat model, grouping, EA intervention, sample collection, and subsequent analyses such as histology, immunohistochemistry, TUNEL assay, western blotting, ELISA, and flow cytometry.

According to previous studies and our preliminary experiments [21], rats in the DOR, EA, and SA groups received an intraperitoneal injection of CTX at 50 mg/kg on the first day of modeling, followed by 5 mg/kg/day for 14 consecutive days. EA and SA interventions were initiated on day 15 of the experiment. The experimental workflow is shown in Figure 1.

### 2.2. Intervention Methods

During acupuncture treatment, rats were gently restrained in a custom‐designed device that immobilized the trunk while leaving the head and limbs free. Acupoints were selected according to the Animal Acupuncture Atlas (Experimental Acupuncture Branch, Chinese Acupuncture and Moxibustion Society). CV4 and CV6 were located on the ventral midline of the lower abdomen in rats. CV6 was positioned at the inferior 3/10 point along the line connecting the umbilicus to the pubic symphysis, whereas CV4 was positioned at the inferior 3/5 point along the same line. Rats were placed in the supine position, and acupoints were identified using consistent surface landmarks with standardized distances across animals to ensure reproducibility. Disposable sterile silver needles (Hwato, China; 0.25 mm × 13 mm) were inserted 3–5 mm into CV4 and CV6 until slight muscle twitching was observed and retained for 20 min daily over 14 days. In the SA group, needles were inserted at non‐acupoints located 5 mm lateral to CV4 and CV6 at the same depth and retained under identical conditions. For EA treatment, needles at CV4 and CV6 were connected to the positive and negative outputs of an SDZ‐III EA device (Hwato, China) to deliver a dense–disperse waveform (2/100 Hz) for 20 min once daily. Stimulation intensity was increased gradually from 0.5 mA to the minimal level that produced slight local muscle twitching, and was then maintained at ~1–2 mA (maximum 2 mA), with minor adjustments according to individual impedance and tolerance. Rats in the NOR and DOR groups underwent the same restraint procedure without needle insertion.

At the end of the intervention, rats in the diestrus phase were identified by vaginal smear cytology, anesthetized with intraperitoneal 2% pentobarbital sodium (0.2 mL/100 g), and then blood, bilateral ovaries, and spleens were collected for further analysis.

### 2.3. Estrous Cycle Monitoring

Vaginal smears were collected daily between 8:00 and 10:00 a.m. using a sterile saline lavage. Smears were air‐dried, stained with Crystal Violet Ammonium Oxalate Solution (Solarbio, China), and examined under a light microscope to determine the estrous cycle stage based on the proportion of epithelial cells, cornified cells, and leukocytes.

### 2.4. Body, Ovarian, and Splenic Weights

All animals were weighed prior to model induction, and body weight was monitored every 2 days throughout the experiment. At the end of treatment, final body weight was recorded, and the ovaries and spleens were collected and weighed.

### 2.5. Serum Hormone and Cytokine Assays

Serum samples were stored at −80°C until analysis. The levels of AMH (JL12462, Jianglai Biology, China), FSH (JL13251, Jianglai Biology, China), LH (JL11706, Jianglai Biology, China), E2 (JL11525, Jianglai Biology, China), TGF‐β (JL13643, Jianglai Biology, China), IL‐6 (JL20896, Jianglai Biology, China), IL‐10 (JL13427, Jianglai Biology, China), and IL‐17 (JL20879, Jianglai Biology, China) were measured by rat‐specific ELISA kits in compliance to manufacturer’s protocols, with their concentrations quantified based on the 450 nm absorbance.

### 2.6. Hematoxylin–Eosin (H&E)

Ovarian tissues were fixed in 4% paraformaldehyde, dehydrated, embedded in paraffin, and sectioned at 5 μm thickness. Sections were deparaffinized, rehydrated, and stained with HE (Wuhan Xavier Biotechnology Co., Ltd., Wuhan, China). After dehydration and mounting, ovarian morphology was examined under a light microscope. Follicles at different developmental stages—including primordial, primary, secondary, antral, and atretic follicles—as well as corpora lutea, were quantified by two independent investigators in a blinded manner. Follicle classification was based on established morphological criteria: primordial follicles were identified by an oocyte surrounded by a single layer of flattened follicular cells; primary follicles were defined by an enlarged oocyte surrounded by a single layer of cuboidal granulosa cells; secondary follicles exhibited an oocyte enclosed by two or more layers of cuboidal granulosa cells with no visible antral cavity; antral follicles were characterized by the presence of multiple layers of granulosa cells, an oocyte with a visible nucleus, a defined antrum, and a theca cell layer; corpora lutea were identified as follicular remnants with hypertrophic and hyperplastic granulosa cells after ovulation.

### 2.7. Western Blotting (WB)

Protein samples were extracted from ovarian and splenic tissues, BCA quantified by (EC0001, SparkJade, China), separated by SDS‐PAGE, and transferred onto PVDF membranes. After blocking, membranes were incubated overnight at 4°C with the following primary antibodies: anti‐GAPDH (1:100000, A19056, ABclonal, China), β‐Tubulin (1:5000, 10094‐1‐AP, Proteintech, China), GDF9 (1:3000, A2739, ABclonal, China), BMP15 (1:1000, ET7110‐03, Huabio, China), Bcl‐2 (1:1000, A0208, ABclonal, China), Bax (1:500, GB11690, Xavier, China), Caspase‐3 (1:1000, bs‐0081R, Bioss, China), FOXP3 (1:1000, ET1702‐12, Huabio, China), RORγt (1:1000, bs‐10647R, Bioss, China), IL‐10 (1:1000, A12255, ABclonal, China), TGF‐β1 (1:3000, A2124, ABclonal, China), TNF‐α (1:1000, A11534, ABclonal, China), IL‐6 (1:500, GB11117, Xavier, China), IL‐17A (1:500, ER1902‐37, Huabio, China), and IL‐1β (1:1000, HA601036, Huabio, China). If the molecular weights of the target proteins were close to that of the loading control, membranes were stripped using Fast stripping buffer (PS107, EpiZyme, China) according to the manufacturer’s instructions before reprobing.

Following primary antibody incubation, membranes were washed three times with TBST and incubated with HRP‐conjugated secondary antibodies (AS014, ABclonal, China) for 1 h at room temperature. After washing, immunoreactive bands were visualized using an enhanced chemiluminescence (ECL) detection kit (ED0025‐A, SparkJade, China) and captured with a ChemiDoc imaging system (Tanon‐4800multi, China). Band intensities were quantified using ImageJ software.

### 2.8. TUNEL Assays

Apoptotic cells in ovarian tissue sections were detected using a TUNEL apoptosis detection kit (C1091, Beyotime Biotechnology, China) following the manufacturer’s instructions. Briefly, paraffin‐embedded ovarian sections were deparaffinized, rehydrated, and treated with proteinase K. After incubation with the TUNEL reaction mixture at 37°C for 60 min in the dark, nuclei were counterstained with DAPI. Images were captured under a fluorescence microscope, and TUNEL‐positive cells were quantified using ImageJ software.

### 2.9. Immunohistochemistry (IHC) Staining

Ovarian tissue sections were deparaffinized, rehydrated, and subjected to antigen retrieval in citrate buffer (pH 6.0). Endogenous peroxidase activity was quenched with 3% H_2_O_2_, and nonspecific binding was blocked with 5% bovine serum albumin (BSA). Sections were incubated overnight at 4°C with primary antibodies against GDF9 (Rabbit pAb, 1:100, bs‐1795R, Bioss, China), FSHR (Rabbit pAb, 1:500, GB11275‐1, Xavier, China), Ki67 (Rabbit pAb, 1:700, GB111499, Xavier, China), or cleaved Caspase‐3 (Rabbit pAb, 1:700, GB11532‐100, Xavier, China). After washing, sections were incubated with HRP‐conjugated secondary antibodies at room temperature for 1 h. Immunoreactivity was visualized using DAB substrate, and nuclei were counterstained with hematoxylin. Images were captured with a light microscope, and staining intensity was quantified using ImageJ software.

### 2.10. Flow Cytometry

Single‐cell suspensions were prepared from splenic tissues and washed with PBS containing 2% fetal bovine serum. For intracellular staining, cells were fixed and permeabilized using the Intracellular Fixation/Permeabilization Buffer Kit (E‐CK‐A109, Elabscience, China). Treg/Th17 populations were detected using the Foxp3/Transcription Factor Staining Kit (E‐CK‐A108, Elabscience, China) in combination with FOXP3 (12‐5773‐80, eBioscience, USA), IL‐17A (25‐7177‐80, eBioscience, USA), and CD4 (11‐0040‐81, eBioscience, USA) monoclonal antibodies. Cells were incubated with the antibodies for 30 min at 4°C in the dark, washed, and resuspended in PBS for analysis on a NovoCyte flow cytometer (Agilent, USA). Treg cells were defined as CD4^+^FOXP3^+^ cells, and Th17 cells were defined as CD4^+^IL‐17A^+^ cells.

### 2.11. Immunofluorescence (IF)

Ovarian tissue sections were deparaffinized, rehydrated, and subjected to antigen retrieval in citrate buffer (pH 6.0). After blocking with 5% BSA for 30 min at room temperature, sections were incubated overnight at 4°C with primary antibodies against FOXP3 (1:500, bs‐0269R, Bioss, China) or RORγt (1:200, bs‐10647R, Bioss, China). After washing, sections were incubated with appropriate fluorophore‐conjugated secondary antibodies for 1 h at room temperature in the dark. Nuclei were counterstained with DAPI, and images were captured using a fluorescence microscope.

### 2.12. RNA Sequencing

Total RNA was isolated and purified from ovarian tissues using TRIzol reagent (Invitrogen, CA, USA) according to the manufacturer’s instructions. RNA concentration and purity were assessed using a NanoDrop ND‐1000 spectrophotometer (NanoDrop, Wilmington, DE, USA), and RNA integrity was evaluated with an Agilent 2100 Bioanalyzer (Agilent, CA, USA) and further confirmed by agarose gel electrophoresis. Library preparation and high‐throughput sequencing were subsequently performed by Lianchuan Biotechnology Co., Ltd. (Hangzhou, China) following standard protocols. Sequencing data were processed for quality control, alignment, and differential gene expression analysis. DEGs were visualized using volcano plots and heatmaps. Functional enrichment analyses, including Gene Ontology (GO) and Kyoto Encyclopedia of Genes and Genomes (KEGG) pathway analyses, were performed to identify biological processes and signaling pathways associated with the DEGs.

### 2.13. Statistical Analysis

Data are presented as mean ± standard deviation (SD). Comparisons among the four groups were performed using one‐way ANOVA followed by Tukey’s post hoc test. A *p*‐Value < 0.05 was considered statistically significant. Analyses were conducted using GraphPad Prism 9.0 and SPSS 26.0.

## 3. Results

### 3.1. EA Improves Estrous Cyclicity, Body Weight, and Organ Indices in DOR Rats

To evaluate the general physiological status of rats, we monitored estrous cycles, body weight, ovarian weight, and spleen weight across the four groups. Vaginal smear analysis revealed that NOR displayed regular 4–5 day estrous cycles, while DOR rats exhibited prolonged diestrus phases and irregular cycles, confirming successful model establishment. EA treatment partially restored estrous cyclicity, whereas SA treatment had no effect (Figure 2A,B).

Figure 2EA improves estrous cyclicity, body weight, and organ weights in DOR rats. (A) Representative schematic of rat estrous cycle stages. (B) Estrous cycle patterns in NOR, DOR, EA, and SA groups. (C) Changes in body weight across the four groups during the experimental period. (D) Ovarian weight. (E) Spleen weight. Data are presented as mean ± SD.

**Figure:**
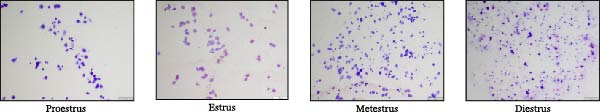
(A)

**Figure:**
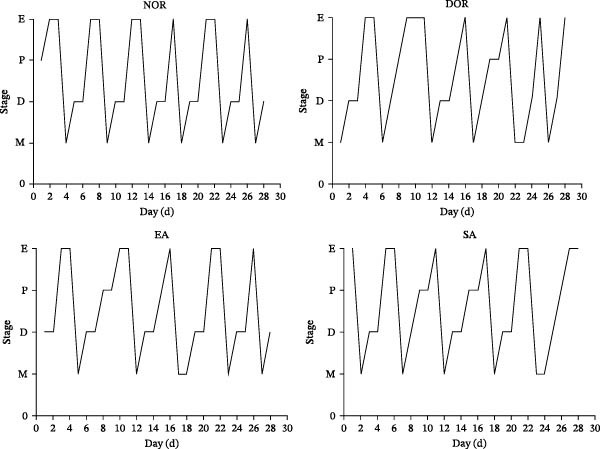
(B)

**Figure:**
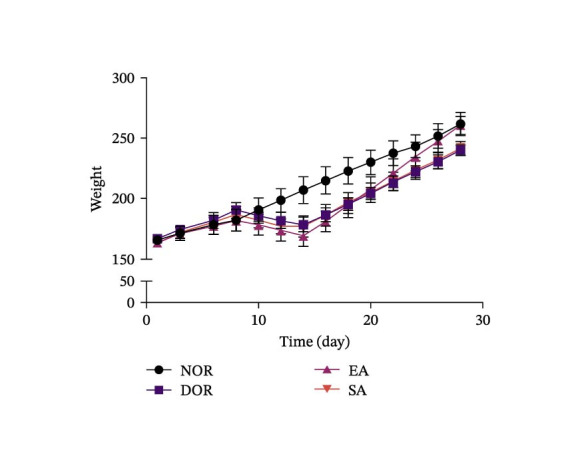
(C)

**Figure:**
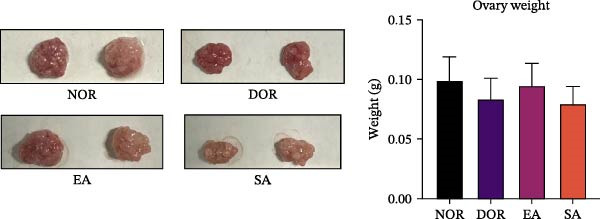
(D)

**Figure:**
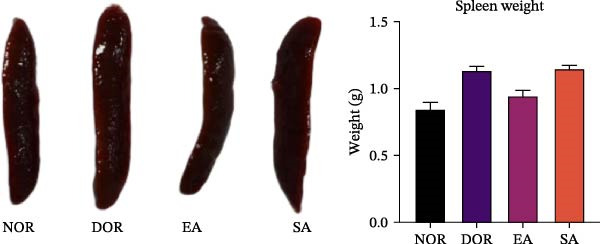
(E)

No differences in body weight were observed among groups prior to CTX injection. After modeling, body weight in the DOR, EA, and SA groups decreased markedly, followed by gradual recovery. By the end of the experiment, the EA group achieved body weight nearly comparable to the NOR group, whereas the DOR and SA groups remained significantly lower (Figure 2C). Ovarian weight was significantly reduced in the DOR group, consistent with impaired ovarian function. EA treatment partially restored ovarian weight compared with the DOR group, while no significant improvement was observed in the SA group (Figure 2D). Similarly, spleen weight was markedly reduced in the DOR group, reflecting systemic immune impairment. EA alleviated this reduction, whereas the SA group showed no significant effect (Figure 2E).

### 3.2. EA Improves Ovarian Follicle Development and Morphology in DOR Rats

Histological analysis revealed marked alterations in follicle development among the groups (Figure 3A,B). Quantification showed that the numbers of primordial, secondary, antral follicles, and corpus luteum were significantly decreased in the DOR group compared with the NOR group. EA treatment significantly increased these follicle counts relative to DOR, whereas SA treatment showed no improvement. Primary follicles were comparable between NOR and DOR, and EA had no significant effect. In contrast, atretic follicles were markedly elevated in the DOR group, while EA significantly reduced follicular atresia.

Figure 3EA improves follicle development and reduces follicular atresia in DOR rats. (A) Quantification of follicles at different developmental stages, including primordial, primary, secondary, and antral follicles, as well as atretic follicles and corpus luteum. Data are presented as mean ± SD. ANOVA test,  ^∗^ *p*  < 0.05,  ^∗∗^ *p*  < 0.01,  ^∗∗∗^ *p*  < 0.001. (B) Representative HE‐stained ovarian sections from the NOR, DOR, EA, and SA groups, showing follicle morphology at different magnifications (500, 100, and 50 μm).

**Figure:**
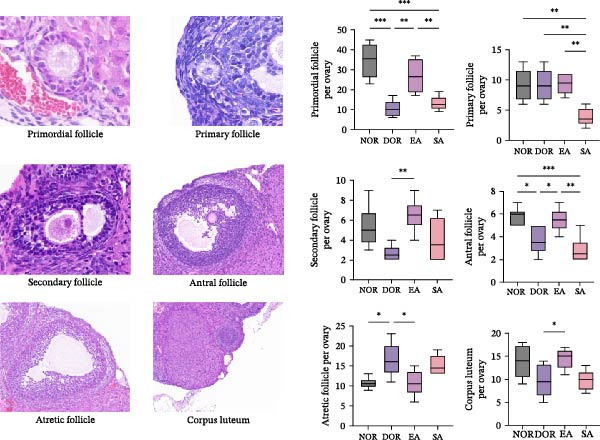
(A)

**Figure:**
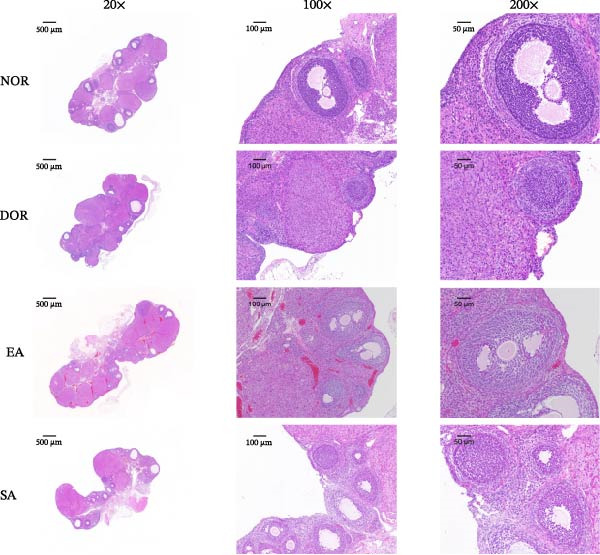
(B)

Representative HE‐stained sections confirmed these findings. NOR rats showed normal ovarian architecture with abundant follicles at various stages, while DOR rats exhibited reduced follicle numbers, increased atresia, and disrupted morphology. EA alleviated these pathological changes, preserving follicular morphology with more growing follicles and fewer atretic follicles. The SA group closely resembled the DOR group.

### 3.3. EA Modulates Serum Hormone Levels and Follicle‐Associated Factors in DOR Rats

As shown in Figure 4A, serum AMH and E2 levels were significantly decreased in the DOR group compared with NOR, and remained low in the SA group. Compared with the DOR group, serum E2 and AMH levels showed upward trends in the EA group, but neither difference reached statistical significance (E2: mean difference = 4.746, 95% CI −0.1979 to 9.690, adjusted *p* = 0.06; AMH: mean difference = 3.066, 95% CI −0.08337 to 6.215, adjusted *p* = 0.06). Conversely, LH, FSH, and the LH/FSH ratio were significantly elevated in the DOR group and remained abnormal in the SA group, whereas EA partially alleviated these endocrine disturbances.

Figure 4EA modulates serum hormone levels and follicle‐associated factors in DOR rats. (A) Serum hormone levels of AMH, E2, LH, FSH, and the LH/FSH ratio across groups. DOR rats showed decreased AMH and E2 and increased LH, FSH, and LH/FSH ratio compared with NOR. EA showed upward trends in AMH and E2 and reduced gonadotropin elevation, whereas SA resembled DOR. Data are presented as mean ± SD. One‐way ANOVA,  ^∗^ *p*  < 0.05,  ^∗∗^ *p*  < 0.01,  ^∗∗∗^ *p*  < 0.001. (B) Western blot analysis of follicle‐associated proteins BMP15 and GDF9 in ovarian tissue. EA restored their expression, which was reduced in DOR. Data are presented as mean ± SD. One‐way ANOVA,  ^∗^ *p*  < 0.05,  ^∗∗^ *p*  < 0.01,  ^∗∗∗^ *p*  < 0.001. (C) Immunohistochemistry for FSHR and GDF9 expression in ovarian tissue. DOR and SA groups showed reduced expression compared with NOR, while EA reversed these changes. Scale bar = 50 μm. Data are presented as mean ± SD. One‐way ANOVA,  ^∗^ *p*  < 0.05,  ^∗∗^ *p*  < 0.01,  ^∗∗∗^ *p*  < 0.001.

**Figure:**
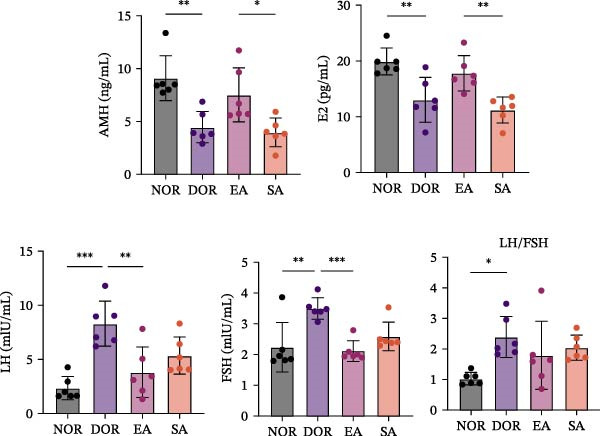
(A)

**Figure:**
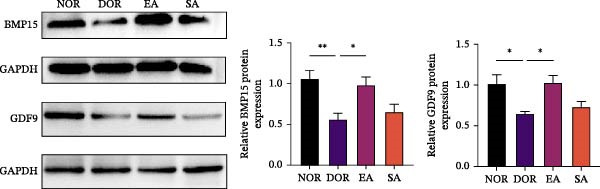
(B)

**Figure:**
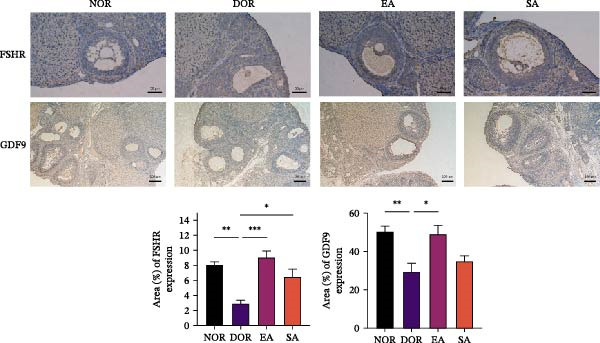
(C)

Western blot analysis revealed significantly reduced BMP15 and GDF9 expression in the DOR group compared with NOR, whereas EA restored their expression (Figure 4B). Consistently, IHC confirmed downregulation of FSHR and GDF9 in the DOR group, which was reversed by EA (Figure 4C). SA treatment produced no improvement, remaining similar to DOR. Together, these findings indicate that EA partially ameliorates endocrine disturbances, particularly gonadotropin abnormalities, while restoring key follicle‐associated factors, whereas SA treatment has negligible effects.

### 3.4. EA Attenuates Granulosa Cell Apoptosis and Restores Proliferative Capacity

TUNEL staining revealed extensive granulosa cell apoptosis in DOR ovaries compared with NOR. EA significantly reduced apoptosis, whereas SA showed no effect (Figure 5A). Ki67 IHC showed reduced proliferative activity in the DOR group, which was restored toward NOR levels by EA, while SA remained unchanged. Western blot analysis further showed decreased Bcl‐2 and increased Bax expression in the DOR group, both reversed by EA. In contrast, SA resembled DOR. IHC also revealed markedly elevated Cleaved Caspase‐3 in DOR, but neither EA nor SA significantly altered its expression (Figure 5B,C). Collectively, these findings suggest that EA attenuated apoptosis‐related changes in granulosa cells, as reflected by reduced TUNEL positivity and normalization of the Bcl‐2/Bax axis, while cleaved caspase‐3 remained unchanged, indicating that the antiapoptotic effect of EA may be partial rather than uniform across all apoptotic endpoints.

Figure 5EA attenuates granulosa cell apoptosis and restores proliferative activity in DOR rats. (A) TUNEL staining of ovarian tissue showing apoptotic granulosa cells (red) and DAPI‐stained nuclei (blue). Ki67 immunohistochemistry (IHC) revealed reduced proliferation in the DOR group, which was restored by EA. Scale bar = 50 μm. Data are presented as mean ± SD. One‐way ANOVA,  ^∗^ *p*  < 0.05,  ^∗∗^ *p*  < 0.01,  ^∗∗∗^ *p*  < 0.001. (B) Western blot analysis of apoptosis‐related proteins (Bcl‐2 and Bax) in ovarian tissue. EA significantly reversed the downregulation of Bcl‐2 and the upregulation of Bax observed in the DOR group. Data are presented as mean ± SD. One‐way ANOVA,  ^∗^ *p*  < 0.05,  ^∗∗^ *p*  < 0.01,  ^∗∗∗^ *p*  < 0.001. (C) IHC analysis of cleaved caspase‐3 expression in ovarian tissue. EA did not significantly alter cleaved caspase‐3 levels compared with the DOR group. Data are presented as mean ± SD. One‐way ANOVA,  ^∗^ *p*  < 0.05,  ^∗∗^ *p*  < 0.01,  ^∗∗∗^ *p*  < 0.001.

**Figure:**
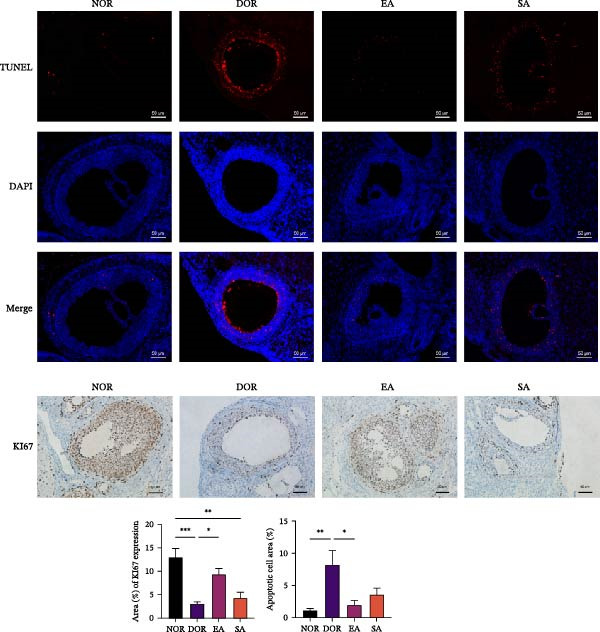
(A)

**Figure:**
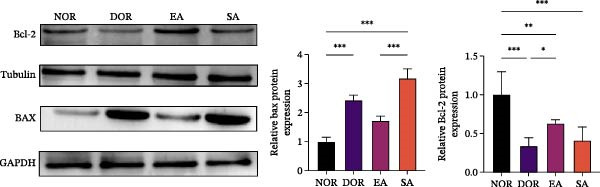
(B)

**Figure:**
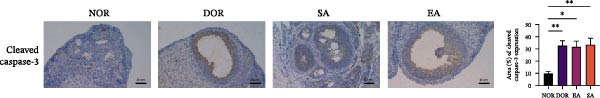
(C)

### 3.5. EA Restores Th17/Treg Immune Balance in the Spleen

To test whether EA exerts systemic immunomodulatory effects, we examined the spleen, a key peripheral immune organ. Flow cytometry revealed reduced Treg cells and increased Th17 cells in DOR rats compared with NOR. EA reversed these alterations, significantly increasing Tregs and decreasing Th17 cells, whereas SA showed no effect (Figure 6A). WB confirmed that Th17‐associated proteins (IL‐6, TNF‐α, IL‐17A, RORγt, and IL‐1β) were markedly elevated in DOR but reduced by EA (Figure 6B). SA showed no improvement. Conversely, Treg‐associated proteins (IL‐10, TGF‐β1, and FOXP3) were downregulated in DOR, restored by EA but unchanged in SA (Figure 6C). These results indicate that EA was associated with restoration of Th17/Treg‐related immune balance in the spleen.

Figure 6EA restores Th17/Treg balance in the spleen of DOR rats. (A) Flow cytometry analysis of Treg/Th17 cell ratio with statistical quantification. Data are shown as mean ± SD. One‐way ANOVA,  ^∗^ *p*  < 0.05,  ^∗∗^ *p*  < 0.01,  ^∗∗∗^ *p*  < 0.001. (B, C) Western blot analysis of Th17‐related proteins (IL‐6, TNF‐α, IL‐17 A, RORγt, and IL‐1β) and Treg‐related proteins (IL‐10, TGF‐β1, and FOXP3) in spleen tissue. Data are shown as mean ± SD. One‐way ANOVA,  ^∗^ *p*  < 0.05,  ^∗∗^ *p*  < 0.01,  ^∗∗∗^ *p*  < 0.001.

**Figure:**
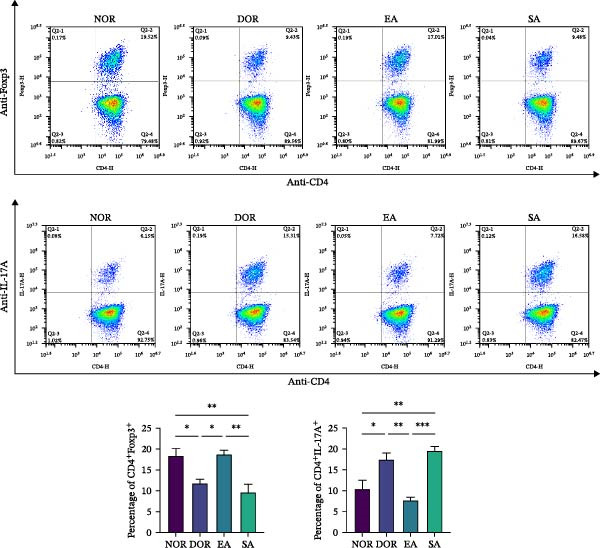
(A)

**Figure:**
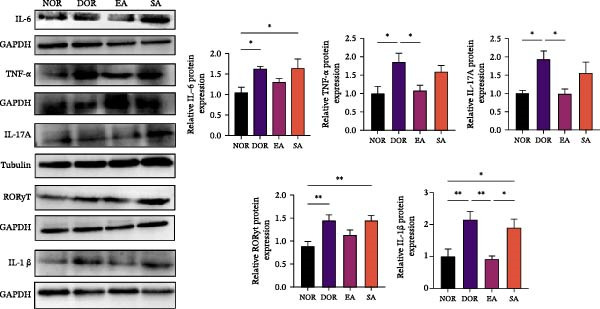
(B)

**Figure:**
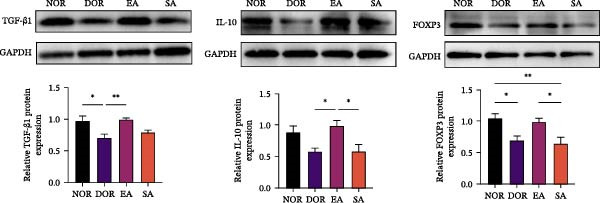
(C)

### 3.6. EA Modulates Th17/Treg‐Related Proteins in Ovarian Tissue

We next examined whether these systemic immune changes were reflected locally in ovarian tissue. IHC showed increased RORγt and decreased FOXP3 expression in the DOR group, both of which were reversed by EA but not by SA (Figure 7A,B).

Figure 7EA restores Th17/Treg balance in ovarian tissue of DOR rats. (A, B) Immunofluorescence staining of FOXP3 and RORγt in ovarian tissue. Scale bar = 50 μm. Data are shown as mean ± SD. One‐way ANOVA,  ^∗^ *p*  < 0.05,  ^∗∗^ *p*  < 0.01,  ^∗∗∗^ *p*  < 0.001. (C, D) Western blot analysis of Th17‐related proteins (IL‐6, TNF‐α, IL‐17A, RORγt, and IL‐1β) and Treg‐related proteins (IL‐10, TGF‐β1, and FOXP3) in ovarian tissue. Data are shown as mean ± SD. One‐way ANOVA,  ^∗^ *p*  < 0.05,  ^∗∗^ *p*  < 0.01,  ^∗∗∗^ *p*  < 0.001.

**Figure:**
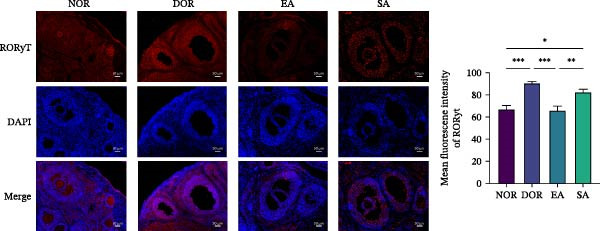
(A)

**Figure:**
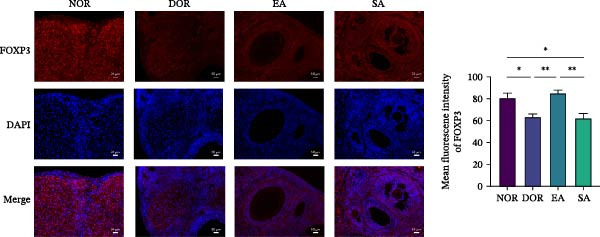
(B)

**Figure:**
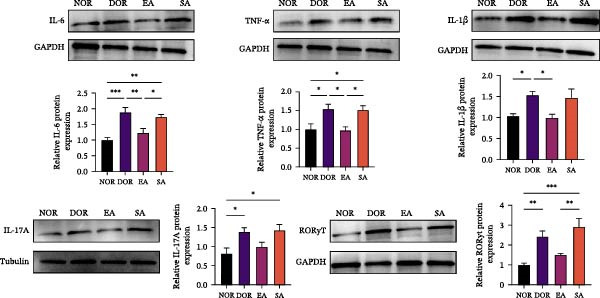
(C)

**Figure:**
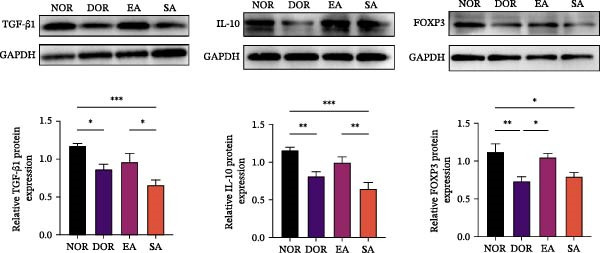
(D)

Western blot analysis further confirmed elevated Th17‐related proteins (IL‐6, TNF‐α, IL‐17A, RORγt, IL‐1β) and reduced Treg‐related proteins (IL‐10, TGF‐β1, FOXP3) in DOR ovaries. EA intervention significantly reversed these alterations, whereas SA resembled DOR (Figure 7C,D).

### 3.7. EA Restores Th17/Treg‐Associated Cytokine Balance in Serum

ELISA assays further validated systemic immune regulation by EA. Th17‐related cytokines IL‐6 and IL‐17 were significantly elevated in DOR serum but decreased after EA, whereas SA showed no improvement. Conversely, Treg‐related cytokines IL‐10 and TGF‐β were significantly reduced in DOR but restored by EA (Figure 8A,B).

Figure 8EA treatment alters serum Treg/Th17‐related cytokine levels in DOR rats. (A) IL‐6 and IL‐17. (B) IL‐10 and TGF‐β. Data are shown as mean ± SD. One‐way ANOVA,  ^∗^ *p*  < 0.05,  ^∗∗^ *p*  < 0.01,  ^∗∗∗^ *p*  < 0.001. (C) Schematic illustration of EA‐mediated regulation of systemic immune balance in DOR rats.

**Figure:**
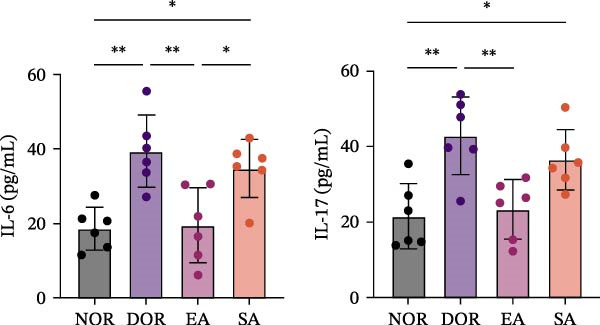
(A)

**Figure:**
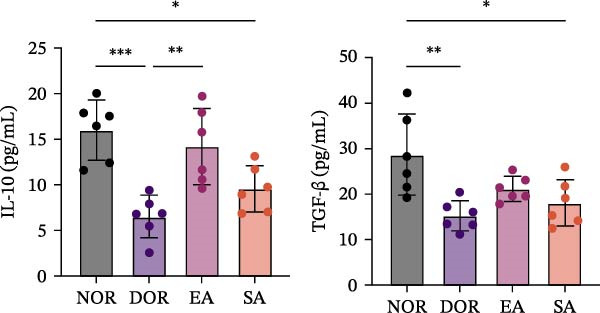
(B)

**Figure:**
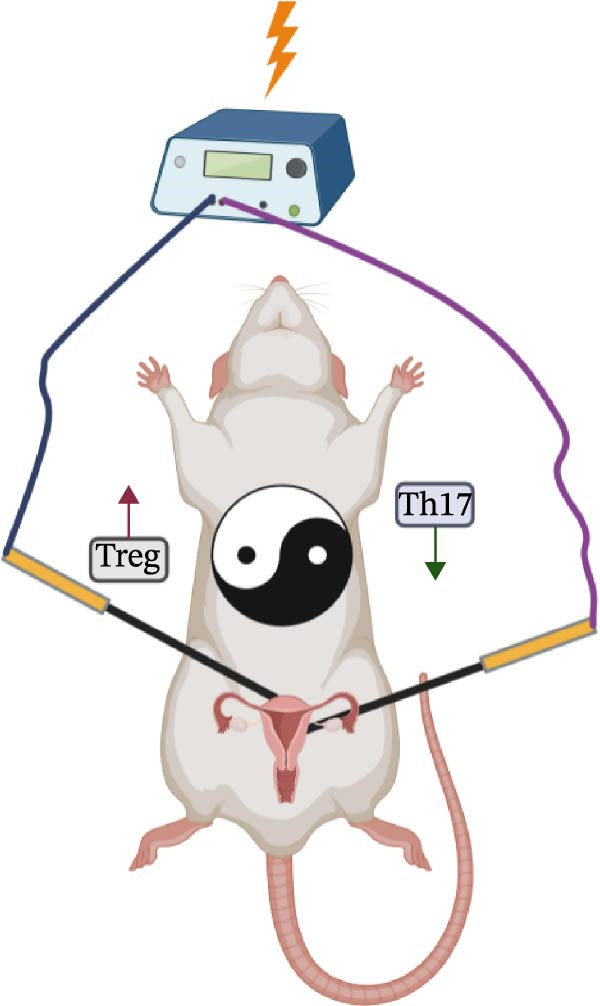
(C)

### 3.8. EA Modulates Immune‐Related Gene Expression and Signaling Pathways

Given the profound effects of EA on immune homeostasis, we next performed transcriptomic analysis of ovarian tissue. Volcano plots and heatmaps demonstrated numerous DEGs and clear separation between groups, indicating robust transcriptomic alterations induced by EA (Figure 9A,B).

Figure 9Transcriptomic analysis reveals that EA modulates immune‐related gene expression and pathways. (A, B) Volcano plots and heatmaps show distinct differential gene expression profiles between EA and DOR groups (*p*  < 0.05, |Log_2_FC| > 1). (C, D) GO enrichment analysis of DEGs. (E, F) KEGG enrichment analysis of DEGs.

**Figure:**
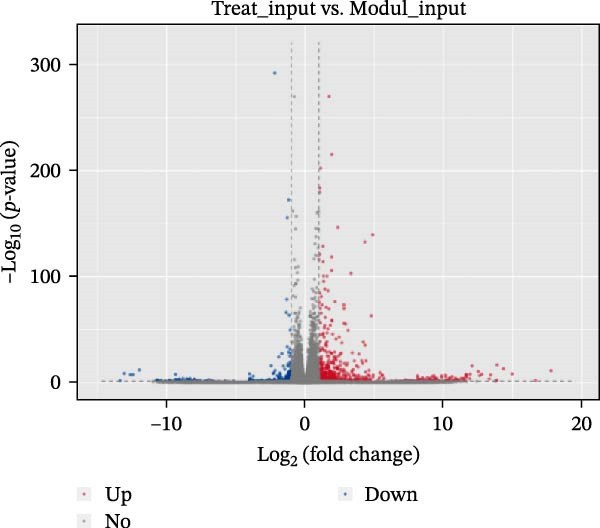
(A)

**Figure:**
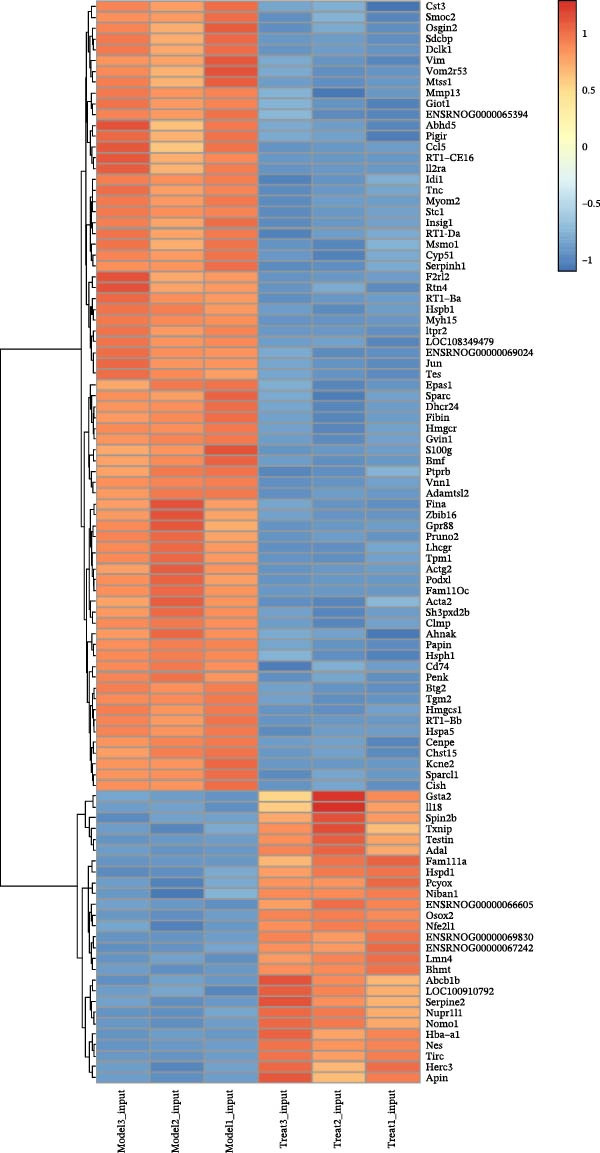
(B)

**Figure:**
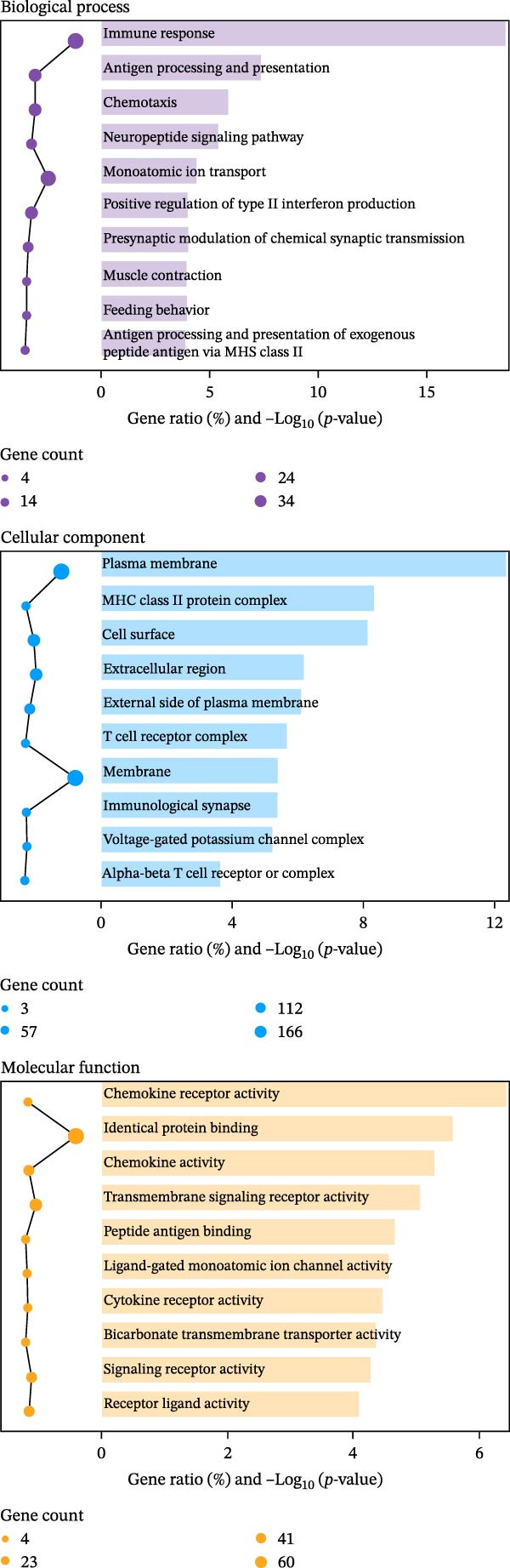
(C)

**Figure:**
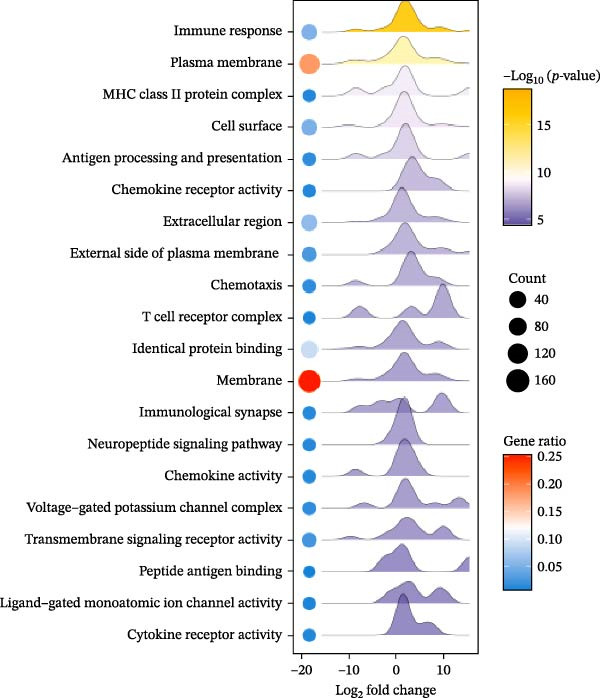
(D)

**Figure:**
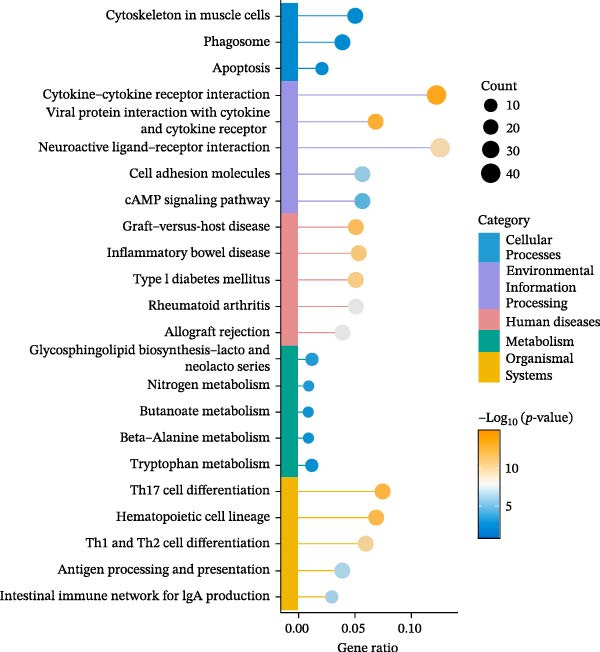
(E)

**Figure:**
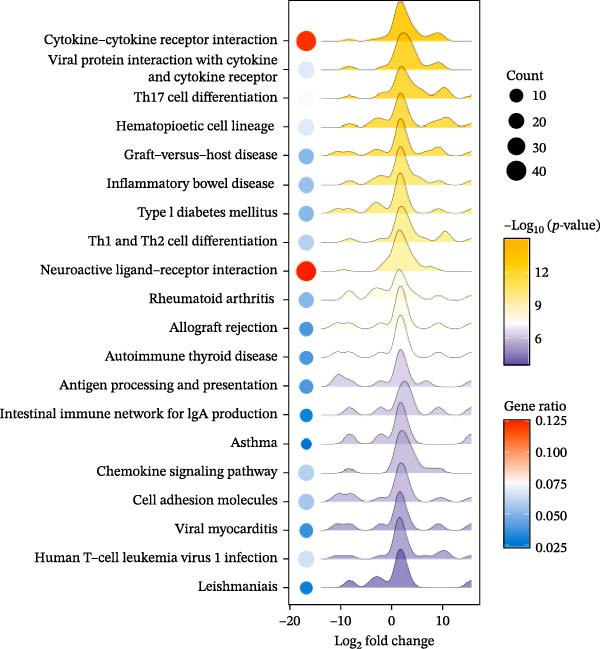
(F)

GO analysis revealed enrichment of DEGs in immune processes such as antigen processing and presentation, T cell receptor signaling, and chemokine receptor activity, with localization to the plasma membrane and MHC complexes (Figure 9C,D). KEGG pathway analysis further showed enrichment in cytokine–cytokine receptor interaction, Th17 cell differentiation, antigen processing and presentation, chemokine signaling, and autoimmune disease pathways, including rheumatoid arthritis and type I diabetes mellitus (Figure 9E,F). Together, these findings demonstrate that EA induces broad transcriptomic changes in ovarian tissue, prominently modulating immune‐related signaling, particularly T cell–mediated pathways, which may underlie its protective effects on ovarian function.

## 4. Discussion

DOR represents a major challenge in reproductive medicine, with current therapeutic options remaining limited and often unsatisfactory in terms of long‐term efficacy [22]. In the present study, EA ameliorated CTX‐induced ovarian injury in rats, as reflected by improved estrous cyclicity, ovarian morphology, follicular development, and recovery of follicle‐associated markers, including BMP15, GDF9, and FSHR. EA also reduced the elevation of gonadotropins observed in the DOR group. However, although AMH and E2 showed upward trends after EA treatment, these changes did not reach statistical significance in the current study. Therefore, the endocrine benefit of EA should be interpreted cautiously as a partial improvement in ovarian dysfunction rather than definitive restoration of ovarian reserve. In parallel, transcriptomic analysis revealed enrichment of immune‐related pathways, supporting the possibility that immunoregulation is involved in the ovarian protective effects of EA.

Granulosa‐cell injury is a critical event in follicular atresia and DOR progression [23]. In this study, DOR ovaries showed increased TUNEL positivity, reduced Ki67 expression, and an unfavorable shift in the Bcl‐2/Bax axis, together with decreased expression of follicle‐supportive proteins. EA significantly reduced TUNEL‐positive cells, restored Ki67 expression, increased Bcl‐2, and decreased Bax, indicating that EA attenuated apoptosis‐related changes and partially preserved granulosa‐cell survival status. Notably, however, cleaved caspase‐3 remained elevated and was not significantly altered by EA. This finding suggests that the protective effect of EA should not be interpreted as a uniform or complete suppression of apoptosis. Rather, EA may preferentially regulate upstream mitochondrial apoptosis‐related signaling and survival homeostasis, instead of fully inhibiting downstream executioner caspase activation. In addition, the discrepancy among apoptosis‐related readouts raises the possibility that classical caspase‐dependent apoptosis may not be the only cell‐death process involved in granulosa‐cell injury in this model. Other forms of regulated nonapoptotic cell death may also contribute to CTX‐induced ovarian damage and to the protective effects of EA; however, because no pathway‐specific markers were assessed in the present study, this possibility should be considered a future research direction rather than a mechanistic conclusion of the current work.

Accumulating evidence indicates that immune imbalance contributes to ovarian aging and follicle depletion, particularly through disruption of the Th17/Treg axis [24–26]. In our study, DOR was characterized by systemic and local immune disequilibrium, including increased Th17‐related cells, cytokines, and proteins, together with reduced Treg‐associated markers in the spleen, serum, and ovary. EA consistently shifted these indices toward a more balanced immune profile, while RNA‐seq further identified enrichment of pathways related to T‐cell signaling, cytokine–cytokine receptor interaction, chemokine signaling, and Th17 differentiation. These convergent findings support a close association between EA‐mediated ovarian protection and Th17/Treg‐related immune modulation. Nevertheless, these data remain correlational. Because no immune‐cell depletion, adoptive‐transfer, or pathway‐blocking experiments were performed, the present study does not establish that Th17/Treg rebalancing is mechanistically required for the ovarian benefits of EA. Accordingly, our results support an immunoregulatory association rather than definitive causal proof.

**Figure 10:**
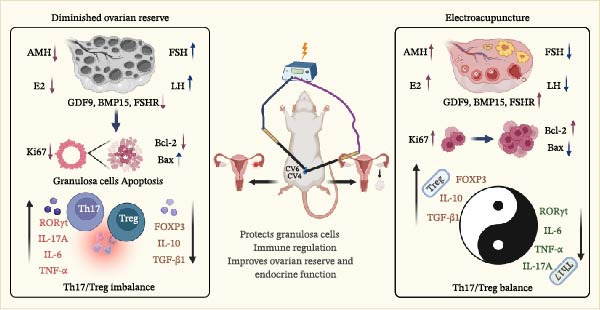
Schematic summary of the observed effects of EA in CTX‐induced DOR rats. EA may ameliorate CTX‐induced ovarian impairment by attenuating apoptosis‐related changes in granulosa cells and by modulating Th17/Treg‐related immune imbalance at systemic and local levels.

Several limitations should be acknowledged. First, although many functional changes were observed in granulosa‐cell‐related readouts, RNA sequencing and WB were performed on whole ovarian tissue, which contains multiple cell populations; thus, cell‐type specificity remains unresolved. Second, the endocrine recovery was incomplete, as AMH and E2 did not show statistically significant restoration. Third, apoptosis‐related endpoints were not uniformly normalized, particularly cleaved caspase‐3, and alternative regulated cell‐death pathways were not directly examined. Fourth, the immune findings were based on associative evidence without interventional validation of mechanistic necessity. Finally, the CTX‐induced rat model captures an important form of ovarian injury but cannot fully represent the etiologic heterogeneity and chronic course of human DOR. Despite these limitations, our findings suggest that EA may serve as a promising nonpharmacological adjunct for ameliorating ovarian dysfunction, preserving follicular microenvironment homeostasis, and modulating immune imbalance in DOR. Future studies should incorporate purified granulosa‐cell models, cell‐type‐resolved analyses, immune‐intervention experiments, and direct validation of additional regulated cell‐death pathways, as well as well‐designed clinical studies in women with DOR. A schematic summary of the proposed EA‐associated protective model is shown in Figure 10.

## 5. Conclusion

EA ameliorated ovarian injury in CTX‐induced DOR rats and was associated with improved follicular development, attenuation of apoptosis‐related changes, and Th17/Treg‐related immune modulation. These findings support EA as a promising nonpharmacological strategy for DOR, while further studies are needed to verify endocrine efficacy and mechanistic causality.

## Author Contributions

Xiaoyu Zhang conceived and designed the study and performed the majority of the experiments. Zhanyu Lin carried out the animal experiments. Ruixin Liu and Zhengqi Guo organized and analyzed the experimental data. Kexiang Wang prepared the schematic diagrams. Yuxia Ma was responsible for manuscript revision and submission.

## Funding

The authors declare that financial support was received for the research, authorship, and publication of this article. This work was supported by the National Key Research and Development Program Funding Project (Grant 2022YFC3500403), the NATCM’s Project of High‐level Construction of Key TCM Disciplines (Grant zyyzdxk‐2023116), and Quality improvement and innovation project of doctoral students in Shandong University of Traditional Chinese Medicine (Grant YJSTZCX2024003).

## Disclosure

All authors read and approved the final manuscript.

## Consent

The authors have nothing to report.

## Conflicts of Interest

The authors declare no conflicts of interest.

## Data Availability

The data that support the findings of this study are available from the corresponding author upon reasonable request.
